# Obinutuzumab in Rituximab-Intolerant Antineutrophil Cytoplasmic Antibody–Associated Vasculitis Patients

**DOI:** 10.1016/j.ekir.2025.01.022

**Published:** 2025-01-19

**Authors:** Jolijn R. van Leeuwen, Obbo W. Bredewold, Ton J. Rabelink, Y.K.Onno Teng

**Affiliations:** 1 Center of Expertise for Lupus-, Vasculitis- and Complement-Mediated Systemic Diseases, Department of Internal Medicine - Nephrology Section, Leiden University Medical Center, Leiden, The Netherlands

## Introduction

Anti-CD20 therapy with rituximab is the basis for both induction and maintenance treatment in antineutrophil cytoplasmic antibody(ANCA)–associated vasculitis (AAV).^1^ However, some patients experience serious infusion or allergic reactions related to the development of human antichimeric antibodies. Human anti-chimeric antibodies are frequently observed during the treatment of autoimmune diseases (up to 38%) but are understudied in AAV.^2^ Rituximab-intolerant patients with AAV are forced to rely on alternatives such as azathioprine, which have a lower efficacy and are less cost-effective.^1^

A logical, within-class alternative to rituximab is obinutuzumab, for which equivalent or even superior effectiveness can be expected. Obinutuzumab is a novel, humanized type 2 antiCD20 monoclonal antibody with demonstrated superiority to rituximab for treating lymphocytic leukemia and follicular lymphoma.^3^ In addition, superior *in vitro* B-cell cytotoxicity was demonstrated for rheumatoid arthritis and systemic lupus erythematosus.^4^ Recently, obinutuzumab showed favorable clinical outcomes without safety concerns as add-on therapy in a phase II trial for lupus nephritis.^5^

However, data on the effectiveness of obinutuzumab in patients with AAV are lacking. Until now, only 1 small case series has described the clinical efficacy without safety concerns for repeated obinutuzumab treatments in 3 patients with AAV.^6^ Moreover, there are no reports on immunological effects, such as time to B-cell repopulation in AAV or any autoimmune disease. This information is pivotal in determining the correct dosing strategy for obinutuzumab in AAV, which can significantly affect its safety and cost-effectiveness. Herein, we report the immunological effects of obinutuzumab therapy in a cohort of patients with rituximab-intolerant AAV.

## Results

Six patients with AAV (4× microscopic polyangiitis and 2× granulomatosis with polyangiitis) who previously responded to rituximab but developed rituximab intolerance were studied in a case-control setting, where the patients were their own controls (Table 1). In these patients, ANCA titers and B-cell measurements were analyzed and compared between rituximab and obinutuzumab treatments. The detailed methods are provided in the Supplementary Methods. Nine obinutuzumab cycles (4 induction treatments and 5 maintenance treatments) and 8 previous rituximab cycles (5 induction treatments and 3 maintenance treatments) were included in the analysis. Importantly, during follow-up with obinutuzumab retreatment (22.6 [14.7–25.8] months), no immunosuppressants other than corticosteroids were administered, no serious infections were observed, and only 1 major relapse occurred. This relapse developed in a patient more than 2 years after obinutuzumab treatment, while B-cells had repopulated for almost 1 year and ANCA had recently converted from negative to positive. The relapse was effectively treated with an induction regimen of obinutuzumab and corticosteroids.

**Table 1:** Patient and treatment characteristics

Patient characteristics	Patients (n = 6)
AAV type, n (%)	
MPO + MPA	4 (67)
PR3 + GPA	2 (33)
Organ involvement^a^, n (%)	
Kidney	4 (67)
Lung	5 (83)
Nervous system	3 (50)
ENT	4 (67)
Skin	3 (50)
Eyes	2 (33)

Treatment characteristics	obinutuzumab (*n* = 9)	rituximab (*n* = 8)
Treatment cycles		
Induction 2 × 1000 mg	2	5
Induction 1 × 1000 mg	2	
Maintenance 1 × 1000 mg	5	3
Follow-up time^b^, median (IQR)	22.6 (14.7–25.8)	18.7 (11.7–26.2)
Cumulative dosage per patient in mg, median (IQR)	2000 (1250–2750)	2750 (2125–3750)

AAV, ANCA-associated vasculitis; ANCA, antineutrophil cytoplasmic antibody; ENT, ear nose and throat; GPA, granulomatosis with polyangiitis; IQR, inter quartile range; MPA, microscopic polyangiitis; MPO, myeloperoxidase; PR3, proteinase 3.

a All patients had involvement of at least 1 major organ (kidney, lung or nervous system).

b Follow-up time ends at next B-cell therapy or at time of study analysis (July 2024).

**Figure 1:**
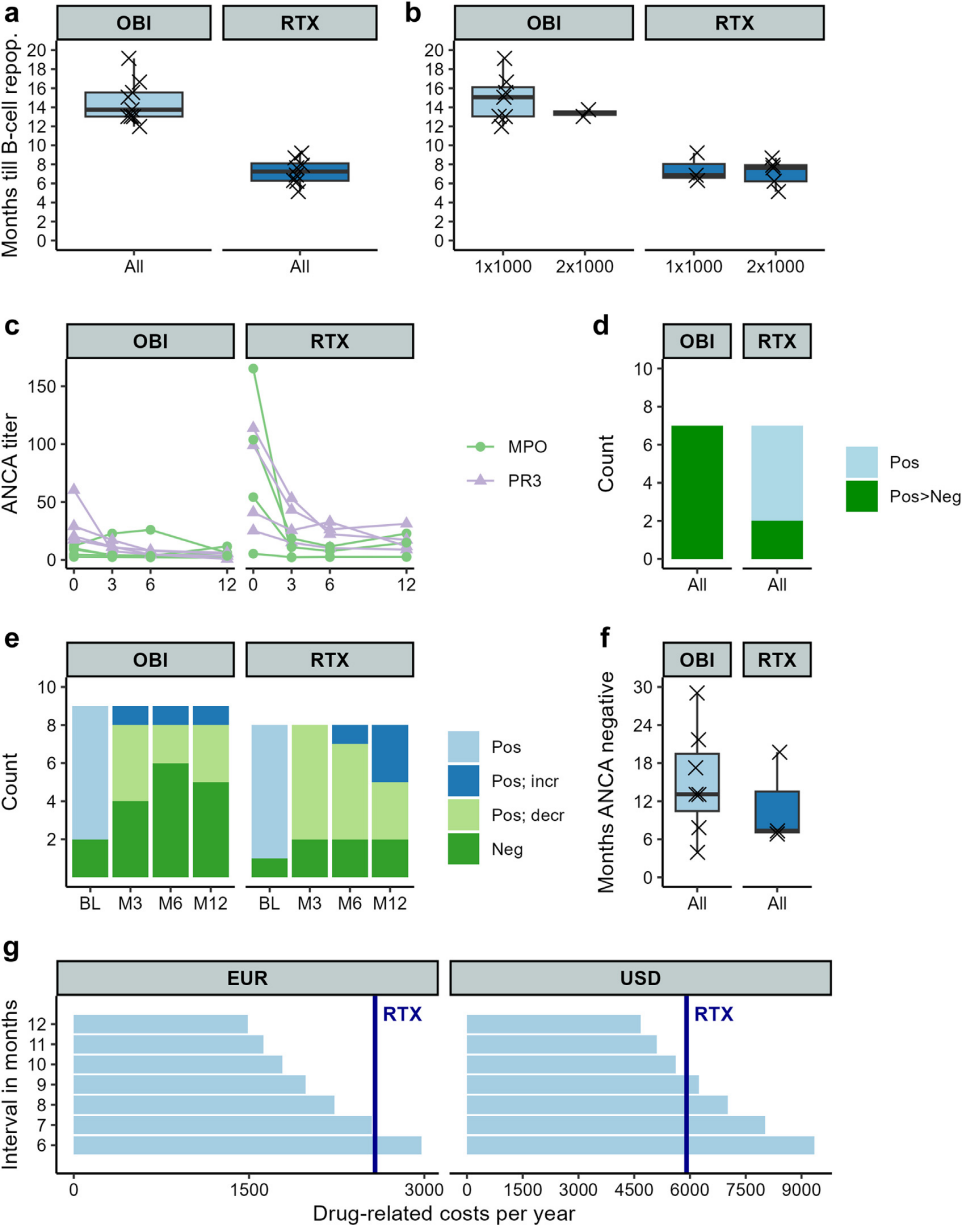
Comparison of immunological effects between obinutuzumab (*n* = 9) (OBI) and rituximab (*n* = 8) (RTX) treatment cycles for (a) months to B-cell repopulation (> 10 × 10^6^ cells/Liter); (b) months to B-cell repopulation subdivided on dosage; (c) individual ANCA courses over 1 year; (d) number of positive ANCA titers that became negative during follow-up; (e) number of negative, positive decreasing, and positive increasing ANCA titers at different timepoints; (f) duration of ANCA negative time in months during follow-up for patients with ANCA negative titers before last follow-up visit (*n* = 7 vs. *n* = 3); and (g) expected drug-related costs in Euros and USD for obinutuzumab 500 mg retreatment with different intervals in months (light blue bars) compared to expected drug-related costs for rituximab 500mg retreatments every 6 months (dark blue line). ANCA, antineutrophil cytoplasmic antibody; decr, decreasing; EUR, Euros; incr, increasing; M, month; MPO, myeloperoxidase; Neg, negative; OBI, obinutuzumab; Pos, positive, PR3, proteinase 3; repop, repopulation; RTX, rituximab; USD, United States dollars.

Comparing immunological outcomes (Figure 1), we confirmed that obinutuzumab led to substantially longer B-cell depletion (13.7 [13.0–15.6] months) than rituximab (7.2 [6.3–8.1] months) (*P* < 0.001). There was no clear relationship between the dosage and the time until B cell repopulation. Baseline ANCA titers were lower for obinutuzumab (12.1 [9.0–20.4]) than for rituximab (76.6 [37.1–106.3]) because of previous rituximab treatment (*P* = 0.01). However, positive ANCA titers more frequently seroconverted to negative during follow-up after obinutuzumab treatment (7/7, 100%) than after rituximab (2/7, 29%) treatment (*P* < 0.01). In addition, more ANCA titers continued to decrease 1 year after the administration of obinutuzumab than after rituximab. Furthermore, the duration of seroconversion to ANCA-negativity was numerically longer after obinutuzumab (13.1 [10.4–19.5] months) than after rituximab (7.3 [7.1–13.5] months) (*P* = 0.52).

Given the observed longer-lasting B cell depletion, we modelled the drug-related costs of obinutuzumab to biosimilar rituximab, with increasing intervals between obinutuzumab retreatments. As such, compared to 6-monthly rituximab (biosimilar) retreatments, obinutuzumab retreatment is cost-effective at an interval of more than 7 months in the European price setting and at an interval of more than 10 months in the American price setting.

## Discussion

This first study on the immunological effects of obinutuzumab in patients with AAV showed superior effects of obinutuzumab compared with rituximab in terms of prolonged B-cell depletion and more frequent ANCA-seroconversion in rituximab-intolerant patients. In addition, we show that obinutuzumab can be a cost-effective alternative for rituximab when increasing retreatment intervals based on the observed prolonged B-cell depletion. Although the optimal retreatment strategy with obinutuzumab should be addressed in a larger randomized study, our data provide reassurance that a within-class switch to obinutuzumab could be considered in rituximab-intolerant patients, and may even be preferred over less efficacious alternatives for maintenance treatment, such as azathioprine.

Importantly, we studied only patients with rituximab-intolerant AAV, which may have influenced the immunological effects measured after rituximab. However, the time to B-cell repopulation after rituximab reported in this study is in line with the median time between tailored rituximab-retreatments reported in the MAINRITSAN2 trial (6.1 [3.1–9.2] months).^7^ Moreover, the same trial reported seroconversion from positive to negative in only 52% of ANCA-positive patients after repeated rituximab treatments, whereas both our study as well as a previous case series of 3 patients with AAV described seroconversion in all patients treated with obinutuzumab.^6^^,^^7^

Although the superiority of obinutuzumab in terms of immunological outcomes is evident, this study was too small to draw any relevant conclusions regarding the clinical outcomes. Nevertheless, both B cell depletion and ANCA seroconversion are well-established biomarkers of long-term sustained clinical remission and reduced risk of major relapses. Therefore, one can speculate that based on immunological outcomes, obinutuzumab could be superior to rituximab in terms of relevant clinical outcomes. However, in the present cases, pretreatment with rituximab might have impacted the immunological outcomes, which warrants confirmation in a large, randomized clinical study.^1^^,^^8^ We eagerly await further corroboration from the phase 2 OBIVAS study, which focused on local and systemic immunological effects after 6 months of obinutuzumab.^9^

In conclusion, our data confirm the expected immunological superiority of obinutuzumab over rituximab in a cohort of patients with rituximab-intolerant AAV. Moreover, we found that obinutuzumab retreatment could potentially be a cost-effective alternative as a new AAV treatment strategy, prompting the need for further studies to determine the optimal strategy for achieving superior clinical efficacy and cost-effectiveness in AAV.

## Disclosure

All the authors declared no competing interests.

## Data Availability Statement

The data that have been used is confidential and therefore will not be shared.

## Supplementary Material

Supplementary File (PDF) Supplementary Methods. Supplementary References. STROBE statement

## Footnotes

Supplementary File (PDF)

**Supplementary Methods.**

**Supplementary References.**
STROBE statement
