# Supplementary material for: Obinutuzumab in Rituximab-Intolerant Antineutrophil Cytoplasmic Antibody–Associated Vasculitis Patients
Source: Kidney Int Rep. 2025 Jan 19;10(4):1288–91. doi: 10.1016/j.ekir.2025.01.022 (PMC12034865; doi:10.1016/j.ekir.2025.01.022)
Supplement: Supplementary File (PDF) — Supplementary Methods. Supplementary References. STROBE statement [file mmc1.pdf]

SUPPLEMENTARY MATERIAL

1. Supplementary Methods

We performed a retrospective analysis on AAV patients who were treated with obinutuzumab because intolerance to rituximab based on an infusion reaction or an allergic reaction. All AAV diagnoses were confirmed by manual review and all patients gave permission for the use of their medical data for healthcare evaluation and/or research.

All rituximab and obinutuzumab treatments, ANCA titers and absolute B-cell measurements where extracted from the electronic patients records using the CTcue data collector (v4.8.1). Follow-up for each treatment ended at the next B-cell treatment or current date. Only treatment cycles where B-cell repopulation (>$10x10^6$ cells/Liter) was measured during follow-up where included. For most patients B-cells were measured every 3-6 months. To prevent over-estimation of the time to B-cell repopulation, repopulation was set at a maximum 3 months after the latest measurement of B-cell depletion. The ANCA titer at baseline was determined based on the latest measurement before treatment. For patients that with ANCA negative titers before last follow-up visit the ANCA negative time during follow-up was calculated in months. Electronic health records were manually screened for concomitant immunosuppressives, serious infections requiring intravenous antibiotic treatment and major relapses.

Drug-related costs were determined based on costs for 500mg drugs and costs for in-hospital drug administration. For an European price setting, costs for the drugs were calculated using the prices per 1000mg medication as listed at medicijnkosten.nl and average drug administration prices listed in an internal price list of the Leiden University Medical Centre (LUMC).[S1] For an American price setting, costs for the drugs were calculated using the prices per 1000mg as listed in the average sale price (ASP)-drug pricing files (January 2024) of Centers for Medicare and Medicaid Services.[S2] Costs for drug administration were based on the costs estimated for rituximab-administrations.[S3] Prices listed before 2024 were corrected for inflation.

2. Supplementary References

S1. Medicijnkosten.nl: Zorginstituut Nederland; 2024 [Available from: https://www.medicijnkosten.nl/.

S2. 2024 ASP Drug Pricing: Centers for Medicare & Medicaid Services; 2024 [Available from: https://www.cms.gov/medicare/payment/part-b-drugs/asp-pricing-files.

S3. Schmier J, Ogden K, et al. Costs of Providing Infusion Therapy for Rheumatoid Arthritis in a Hospital-based Infusion Center Setting. *Clin Ther*. 2017;39(8):1600-17.DOI: 10.1016/j.clinthera.2017.06.007

3. STROBE Statement—checklist of items that should be included in reports of observational studies

	Item No.	Recommendation	Page No.	Relevant text from manuscript
**Title and abstract**	1	(*a*) Indicate the study's design with a commonly used term in the title or the abstract	NA	No abstract included
		(*b*) Provide in the abstract an informative and balanced summary of what was done and what was found	NA	No abstract included
**Introduction**				
Background/rationale	2	Explain the scientific background and rationale for the investigation being reported	2	Introduction
Objectives	3	State specific objectives, including any prespecified hypotheses	2	Introduction
**Methods**				
Study design	4	Present key elements of study design early in the paper	2	Methods
Setting	5	Describe the setting, locations, and relevant dates, including periods of recruitment, exposure, follow-up, and data collection	2	Methods
Participants	6	(*a*) *Cohort study*—Give the eligibility criteria, and the sources and methods of selection of participants. Describe methods of follow-up<br>*Case-control study*—Give the eligibility criteria, and the sources and methods of case ascertainment and control selection. Give the rationale for the choice of cases and controls<br>*Cross-sectional study*—Give the eligibility criteria, and the sources and methods of selection of participants	2	Methods
		(*b*) *Cohort study*—For matched studies, give matching criteria and number of exposed and unexposed<br>*Case-control study*—For matched studies, give matching criteria and the number of controls per case	8	Supplementary Methods
Variables	7	Clearly define all outcomes, exposures, predictors, potential confounders, and effect modifiers. Give diagnostic criteria, if applicable	8	Supplementary Methods
Data sources/ measurement	8*	For each variable of interest, give sources of data and details of methods of assessment (measurement). Describe comparability of assessment methods if there is more than one group	8	Supplementary Methods
Bias	9	Describe any efforts to address potential sources of bias	3	Discussion
Study size	10	Explain how the study size was arrived at	2	Methods

Continued on next page

Quantitative variables	11	Explain how quantitative variables were handled in the analyses. If applicable, describe which groupings were chosen and why	8	Supplementary Methods
Statistical methods	12	(*a*) Describe all statistical methods, including those used to control for confounding	8	Supplementary Methods
		(*b*) Describe any methods used to examine subgroups and interactions	NA	
		(*c*) Explain how missing data were addressed	NA	
		(*d*) *Cohort study*—If applicable, explain how loss to follow-up was addressed<br>*Case-control study*—If applicable, explain how matching of cases and controls was addressed<br>*Cross-sectional study*—If applicable, describe analytical methods taking account of sampling strategy	NA	
		(*e*) Describe any sensitivity analyses	NA	
**Results**				
Participants	13*	(a) Report numbers of individuals at each stage of study—eg numbers potentially eligible, examined for eligibility, confirmed eligible, included in the study, completing follow-up, and analysed	2	Results
		(b) Give reasons for non-participation at each stage	NA	
		(c) Consider use of a flow diagram	NA	
Descriptive data	14*	(a) Give characteristics of study participants (eg demographic, clinical, social) and information on exposures and potential confounders	2, 6	Results, Table 1
		(b) Indicate number of participants with missing data for each variable of interest	NA	
		(c) *Cohort study*—Summarise follow-up time (eg, average and total amount)	6	Table 1
Outcome data	15*	*Cohort study*—Report numbers of outcome events or summary measures over time		
		Case-control study—Report numbers in each exposure category, or summary measures of exposure	6	Table 1
		Cross-sectional study—Report numbers of outcome events or summary measures		
Main results	16	(*a*) Give unadjusted estimates and, if applicable, confounder-adjusted estimates and their precision (eg, 95% confidence interval). Make clear which confounders were adjusted for and why they were included	2-3	Results
		(*b*) Report category boundaries when continuous variables were categorized	NA	
		(*c*) If relevant, consider translating estimates of relative risk into absolute risk for a meaningful time period	NA	

Continued on next page

Other analyses	17	Report other analyses done—eg analyses of subgroups and interactions, and sensitivity analyses	3, 7	Results, Figure 1
**Discussion**				
Key results	18	Summarise key results with reference to study objectives	3	Discussion
Limitations	19	Discuss limitations of the study, taking into account sources of potential bias or imprecision. Discuss both direction and magnitude of any potential bias	3	Discussion
Interpretation	20	Give a cautious overall interpretation of results considering objectives, limitations, multiplicity of analyses, results from similar studies, and other relevant evidence	3	Discussion
Generalisability	21	Discuss the generalisability (external validity) of the study results	3	Discussion
**Other information**				
Funding	22	Give the source of funding and the role of the funders for the present study and, if applicable, for the original study on which the present article is based	4	Disclosures

*Give information separately for cases and controls in case-control studies and, if applicable, for exposed and unexposed groups in cohort and cross-sectional studies.

Note: An Explanation and Elaboration article discusses each checklist item and gives methodological background and published examples of transparent reporting. The STROBE checklist is best used in conjunction with this article (freely available on the Web sites of PLoS Medicine at http://www.plosmedicine.org/, Annals of Internal Medicine at http://www.annals.org/, and Epidemiology at http://www.epidem.com/). Information on the STROBE Initiative is available at www.strobe-statement.org.
